# Verification of Diagnosis in Tuberculosis: A Case Report and Discussion

**DOI:** 10.7759/cureus.1650

**Published:** 2017-09-03

**Authors:** Amanda M Dave, Abed Adelrahman, Vishisht Mehta, Stephen Cavalieri, Renuga Vivekanadan

**Affiliations:** 1 School of Medicine, Creighton University Medical Center; 2 Internal Medicine, Creighton University Medical Center; 3 Pathology, Creighton University Medical Center; 4 Infectious Disease, Creighton University Medical Center

**Keywords:** china, mdr-tb, discordant-susceptibility

## Abstract

Tuberculosis (TB), caused by strains of Mycobacterium tuberculosis complex (M. tuberculosis), is a pulmonary infection that is spread by airborne droplet transmission. The development and spread of drug-resistant strains of M. tuberculosisgreatly jeopardize TB control efforts. We report the case of a previously healthy 43-year-old male, visiting from China, who presented to the emergency department complaining of hemoptysis of 10 days' duration. Cultures were positive for acid fast bacteria and negative for fungi. M. tuberculosis infection was confirmed by a deoxyribonucleic acid (DNA) probe. The patient was initially started on first-line therapy of isoniazid, rifampin, pyrazinamide, and ethambutol, with pyridoxine. His country of origin, China, increased suspicion for drug-resistant tuberculosis. Two weeks later, susceptibility testing of the M. tuberculosis isolate showed resistance to isoniazid, pyrazinamide, and ethambutol. Therapy was subsequently changed to amikacin, linezolid, moxifloxacin, and rifampin. The isolate was subsequently sent to the Center for Disease Control (CDC) for evaluation. Repeat testing showed that the isolate was susceptible to rifampin, pyrazinamide, and ethambutol. The patient was then restarted on his initial anti-TB regimen and was able to return to China.

The main goals for the treatment of TB are to treat the individual patient and to minimize transmission. Clues that point to the possibility of multiple drug resistant tuberculosis (MDR-TB) include contact with a patient with MDR-TB, origin from an endemic region, or failure of therapy with documented supervision. Collaboration with experts was imperative in ensuring appropriate patient care.

## Introduction

Tuberculosis (TB) is caused by strains of Mycobacterium tuberculosis (M. Tuberculosis). TB is a primarily pulmonary infection spread by airborne droplet transmission. The development and spread of drug-resistant strains of M. tuberculosis greatly jeopardize TB control efforts. In the US, 91 cases of MDR-TB were reported in 2014. It is estimated that preventing a single case of MDR-TB would save the US healthcare system more than $250,000 [1]. As such, appropriate testing is imperative in the diagnosis and management of TB.

## Case presentation

We report the case of a 43-year-old male, visiting from China, who presented to the emergency department complaining of hemoptysis of 10 days’ duration. This was associated with mild right-sided pleuritic chest pain and night sweats. The patient denied any shortness of breath, fever, or weight loss. The patient did not report any history of sick contacts. The patient was admitted from the emergency department to the floor. On admission, the patient had a normal physical examination and no significant laboratory abnormalities. Informed consent was obtained. A chest computed tomography scan showed a cavitary lesion with an air-crescent sign in the apical segment of his right lower lobe, suggestive of fungal infection. 

Acid fast bacillus and fungal cultures were performed on three consecutive early morning sputum specimens after admission; all were positive for acid fast bacteria but were negative for fungi. M. tuberculosis infection was confirmed by the DNA probe method. The patient was discharged on first-line therapy with isoniazid, rifampin, pyrazinamide, and ethambutol with pyridoxine. The patient’s country of origin, China, created concern for MDR-TB; as a result, further evaluation of the isolates was performed.

A molecular test for the rpoB gene coding for rifampin resistance was negative. Two weeks later, susceptibility testing of the isolate showed resistance to isoniazid, pyrazinamide, and ethambutol. Therapy was subsequently changed to amikacin, linezolid, moxifloxacin, and rifampin. After discussion with the Center for Disease Control (CDC), the isolate was sent to the CDC for evaluation of resistance genes. Over the following two weeks, the patient’s symptoms were stable. Results from the CDC were negative for resistance genes. Repeat susceptibility testing showed that the strain was susceptible to isoniazid, pyrazinamide, and ethambutol. With these results in hand, the patient was restarted on his original anti-TB regimen. The patient was then able to return to China. It is our suspicion that the initial susceptibility test was contaminated with oral flora or a respiratory tract organism that was resistant to isoniazid, pyrazinamide, and ethambutol.

## Discussion

The main goals of the treatment of TB are to treat the individual patient and to minimize transmission [1]. In non-human immunodeficiency virus (HIV) patients, first-line treatment of active pulmonary TB includes isoniazid, rifampin, pyrazinamide, and ethambutol with pyridoxine for eight weeks followed by isoniazid and rifampin for 16 weeks. TB can be successfully treated if the appropriate antibiotics are administered for the correct length of time [2]. Multiple drug resistant tuberculosis (MDR-TB) is significantly more difficult and expensive to treat than drug susceptible TB [3]. Therefore, the timely detection and confirmation of MDR-TB are crucial. MDR-TB is resistant to at least isoniazid and rifampin. Extremely drug resistant TB (XDR-TB) is also resistant to quinolones and at least one of the injectable drugs (e.g., amikacin, kanamycin, or capreomycin). TB is endemic in China, with 1.4 million cases and 130,000 deaths annually [4-5]. When treating TB patients from high-resistance areas, it is important to consider both MDR-TB and XDR-TB during evaluation and management [4].

Intrinsic resistance to one anti-tuberculosis drug is present from one in 106 to one in 108 organisms. About one in 1014 organisms has intrinsic resistance to two anti-TB drugs. As a result, anti-TB treatment, irrespective of the resistance on testing, involves multiple anti-TB drugs. Clear and accurate resistance testing is the key to selecting the correct treatment and to keeping resistance to a minimum [6]. Clues in the history that may point to the possibility of MDR-TB include a previous history of TB, a short course of treatment, inadequate treatment, and unsupervised therapy. Additional factors include patient contact with a patient with MDR-TB, origin from an endemic region, or failure of therapy with documented supervision. Furthermore, special attention should be paid to TB patients who are immunocompromised, especially in cases of HIV co-infection.

It is important to verify laboratory results if they are questionable. The molecular tests offered by the Center for Disease Control were instrumental in this regard and served to aid in the diagnosis, treatment, and control of TB in this case. 

The incorrect selection of drugs and/or the insufficient duration of therapy frequently leads to MDR-TB. As a result, the treatment of MDR-TB should be done by an expert. Directly observed therapy has also been a key component of keeping resistance rates low; cities that have instituted such programs have seen resistance rates drop [1].

New modalities to identify MDR-TB include line-probe assays, GeneXpert MTB/RIF, and MTBDRplus. Line-probe assays can be used to determine resistance to fluoroquinolones, injectable drugs, and ethambutol. It is used to ‘rule in’ resistance for fluoroquinolones and injectable agents. However, negative tests are not very reliable. GeneXpert MTB/RIF is an automated nucleic acid amplification assay. It detects M. Tuberculosis DNA and the rpoB gene, which demonstrates rifampin resistance [7-8]. It produces results in under two hours, does not require skilled technicians to perform it, nor does it need stringent biosafety conditions. It can also estimate the disease burden via an inverse correlation with the number of reaction cycles required to obtain a positive result. GeneXpert MTB/RIF is endorsed by the World Health Organization (WHO) for the rapid detection of MDR-TB; it can also be applied to smear negative TB. MTBDRplus is an assay that detects mutations responsible for rifampin and isoniazid resistance (rpoB gene - rifampin; katG and inhA gene - isoniazid) [8-9]. The turnaround time is one to two days. However, the assay does provide results in the presence of contamination and in smear negative cases.

While new tests continue to emerge, the smear, the slide, and the microscope are still the backbone of M. Tuberculosis detection. This is especially true in countries with limited resources. Using sputum or other tissue samples, cultures are obtained and evaluated for drug sensitivity [3]. Testing is ideally done in a reference laboratory; this requires skilled technicians to carry out an evaluation. Significant pitfalls to this modality include time needed to culture and the inability to detect resistance.

## Conclusions

Tuberculosis is a primarily pulmonary infection that is endemic to China and has significant potential for morbidity and mortality. For patients from endemic regions, it is important to consider tuberculosis in the differential; this is particularly important if the patient reports contact with infected individuals or presents with symptoms of hemoptysis, night sweats, or weight loss.

If laboratory evaluations are questionable or suspicious, it is imperative to verify the results. Alternatively, repeat testing can be pursued. Furthermore, in some circumstances, it may prove necessary to collaborate with experts to ensure appropriate patient care. The molecular tests offered by the CDC were instrumental in this regard; repeat susceptibility testing and molecular tests for resistance genes were vital in making a proper diagnosis. Once a diagnosis has been confirmed, appropriate management and precautions, if necessary, can be initiated.
